# A case of aggressive atypical anti-GBM disease complicated by CMV pneumonitis

**DOI:** 10.1186/s12882-019-1227-z

**Published:** 2019-01-31

**Authors:** Barbora Sporinova, Susanna A. McRae, Daniel A. Muruve, Marvin J. Fritzler, Samih H. Nasr, Alex C. Chin, Hallgrimur Benediktsson

**Affiliations:** 1 Internal Medicine Residency Program, Cumming School of Medicine, University of Calgary, Calgary, AB Canada; 2 Department of Pathology and Laboratory Medicine, University of British Columbia, Vancouver, BC Canada; 3 Department of Medicine, Cumming School of Medicine, University of Calgary, Calgary, AB Canada; 4 Mitogen Advanced Diagnostics Laboratory, Cumming School of Medicine, University of Calgary, Calgary, AB Canada; 5 Department of Laboratory Medicine and Pathology, Mayo Clinic, Rochester, MN USA; 6 Department of Pathology and Laboratory Medicine, Cumming School of Medicine, University of Calgary, Calgary, AB Canada; 7 Department of Pathology and Laboratory Medicine, Cumming School of Medicine, University of Calgary, Foothills Medical Center, 1403 29 St NW, Calgary, AB T2N 2T9 Canada

**Keywords:** Anti-GBM disease, Pulmonary-renal syndrome, CMV pneumonitis, Hemoptysis, Pulmonary hemorrhage

## Abstract

**Background:**

Anti-glomerular basement membrane (anti-GBM) disease is characterized by circulating IgG glomerular basement membrane antibodies and is clinically expressed as a rapidly progressive crescentic glomerulonephritis (GN), with 30–60% of patients also developing pulmonary hemorrhage. Classically, the renal biopsy shows glomerular crescent formation, bright linear staining of glomerular basement membranes (GBM) for IgG on direct immunofluorescence (IF), and the serologic presence of circulating anti-GBM antibodies. Recently, patients with linear IgG IF staining, undetectable circulating anti-GBM antibodies and glomerular changes atypical for anti-GBM disease have been described as “atypical anti-GBM disease”, with a distinctly more benign clinical course than typical anti-GBM disease. We present a case report of a patient with negative anti-GBM serology but positive linear IgG staining by IF, severe diffuse crescentic and endocapillary proliferative glomerulonephritis, and renal failure, complicated by severe pulmonary hemorrhage after immunosuppression, likely due to cytomegalovirus (CMV) pneumonitis.

**Case presentation:**

A 24-year-old man was admitted to hospital with hemoptysis and renal failure. Investigations for anti-GBM serology by addressable laser bead immunoassay (ALBIA) was negative for anti-GBM antibodies. Renal biopsy showed diffuse endocapillary proliferative glomerulonephritis with membranoproliferative features and diffuse circumferential crescents. Direct IF showed strong linear staining for IgG along GBMs. The patient’s hemoptysis improved with immunosuppression, but 1 month later he was readmitted with gross hemoptysis, which was refractory to further cyclophosphamide, plasma exchange and rituximab. Bronchoalveolar lavage (BAL) and blood work confirmed CMV pneumonitis, and the patient’s hemoptysis resolved with ganciclovir, though he became dialysis dependent.

**Conclusions:**

This case demonstrates an atypical presentation of anti-GBM disease with both crescents and endocapillary hypercellularity and negative serology. The patient is dialysis dependent, unlike most previously described patients with atypical anti-GBM disease. The course was complicated by CMV pneumonitis, which contributed to the severity of the pulmonary manifestations and added diagnostic difficulty.

## Background

Anti-glomerular basement membrane (anti-GBM) disease is a rare disease, characterized by circulating IgG anti-GBM antibodies, a rapidly progressive crescentic glomerulonephritis, with 30–60% of patients also developing pulmonary hemorrhage due to cross-reactivity of antibodies with alveolar basement membranes [1, 2]. The combination of pulmonary hemorrhage and rapidly progressive glomerulonephritis was first described by Dr. Ernest W. Goodpasture in 1919 in the influenza pandemic at the end of World War I. [3] The diagnosis of anti-GBM disease is typically made by immunofluorescence (IF) for IgG (IgG1 dominant) demonstrating linear staining of glomerular basement membranes and focal linear staining of tubular basement membranes, and the serologic presence of anti-GBM antibodies [1, 2, 4, 5]. Recently, atypical cases of anti-GBM disease characterized by negative serology for circulating anti-GBM antibodies and unusual histology have been reported [6–12].

In most patients, the anti-GBM antibodies are directed against the NC1 domain of the alpha-3 chain of type IV collagen, resulting in activation of the complement cascade and tissue injury typical of a type 2 immune response [4, 5]. Light microscopic findings of renal biopsies usually include severe necrotizing and crescentic glomerulonephritis involving the majority of glomeruli, with lesions in the glomerular basement membranes but the absence of significant endocapillary or mesangial proliferation [2, 4, 13]. Serum creatinine levels at diagnosis have been highly correlated with the percentage of crescentic glomeruli seen on biopsy, and patients with serum creatinine over 4 mg/dL (354 μmol/L), oliguria, and over 50% crescents on biopsy rarely recover renal function. [4, 13].

Treatment of anti-GBM glomerulonephritis involves high dose steroids, and cyclophosphamide for inhibition of antibody production and rebound hypersynthesis, along with plasma exchange for removal of circulating antibodies. [2, 4] Rituximab can be used in combination with cyclophosphamide if initial treatment fails [4]. Recurrence of hemoptysis may not necessarily represent disease refractory to treatment, and superimposed infections such as noted by Chan et al. [14] and Hasegawa et al. [15] must be considered before increasing the intensity of immunosuppression.

We present a case of a 24-year-old male with atypical anti-GBM disease, marked by undetectable anti-GBM antibodies and unusual biopsy findings, who developed a relapse of pulmonary hemorrhage. After lack of response to immunosuppression, the hemorrhage was found to be most likely due to a cytomegalovirus (CMV) pneumonitis.

### Case presentation

A 24-year-old male with a history of hypertension, hypothyroidism, morbid obesity, and significant smoking history (both cigarette and marijuana) presented to urgent care with a two-week history of progressive leg edema and hemoptysis. He was found to have nephrotic range proteinuria and acute renal failure with a serum creatinine of 346 μmol/L (no comparison values available). Chest x-rays showed progressive worsening bilateral patchy opacities (Fig. 4) and chest CT showed mild scattered patchy ground-glass parenchymal opacities bilaterally. Serological tests for anti-GBM (ALBIA), anti-neutrophilic cytoplasmic antibodies (ANCA, MPO, PR3), anti-nuclear antibodies, extractable nuclear antigens (including anti-dsDNA), anti-C1q, hepatitis B and C, HIV, and Streptolysin O were all negative. His C3, C4, and Kappa/Lambda free light chain ratio were within normal range.

**Fig. 1:**
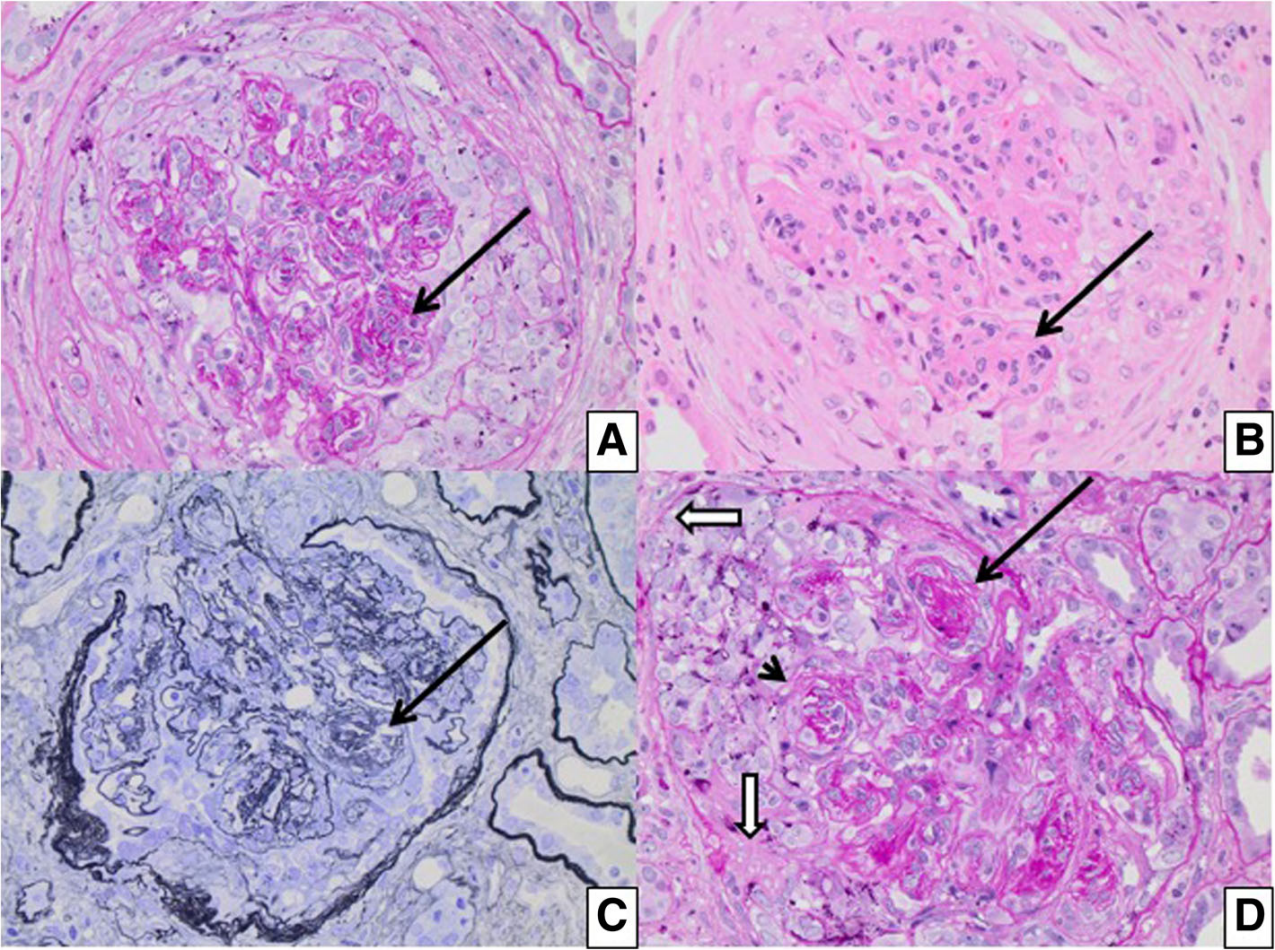
Light microscopic findings: (**a** and **b**) Predominantly cellular crescentic glomerulonephritis with mesangial hypercellularity shown with long arrow (PAS × 400; HE × 400) and (**c** and **d**) nodular sclerosis demarcated with shorter arrow (PASM × 400; PAS × 400). Breaks in Bowman Capsule shown with white arrow and break in GBM with arrowhead. Asterisk denotes focal GBM duplication

**Fig. 2:**
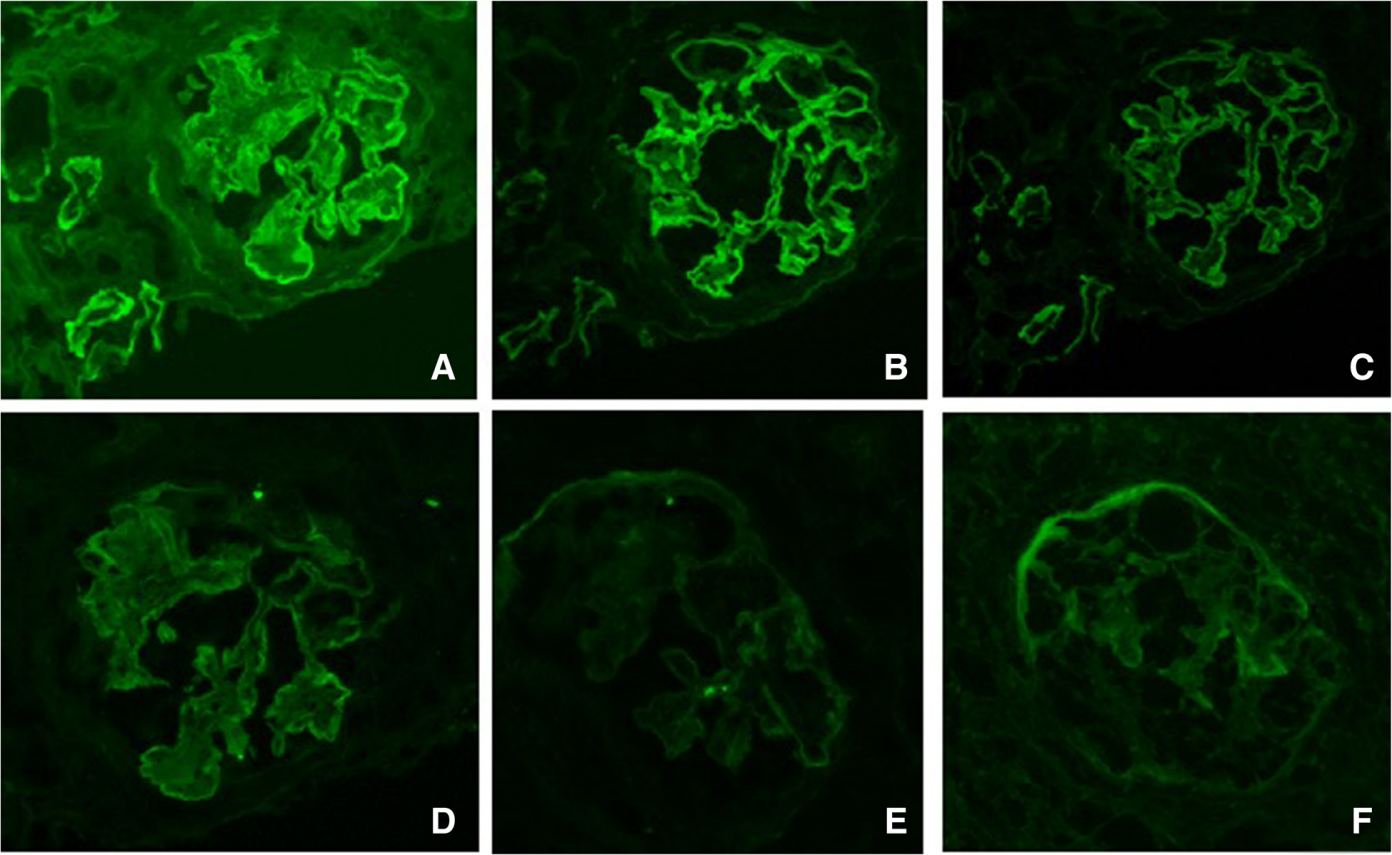
IF shows strong linear staining of GBM and focal TBM for IgG (**a**), lambda (**b**), with weaker staining for kappa (**c**) and IgA (**d**). IgM (**e**) and C3c (**f**) and C1q (not shown) were negative

**Fig. 3:**
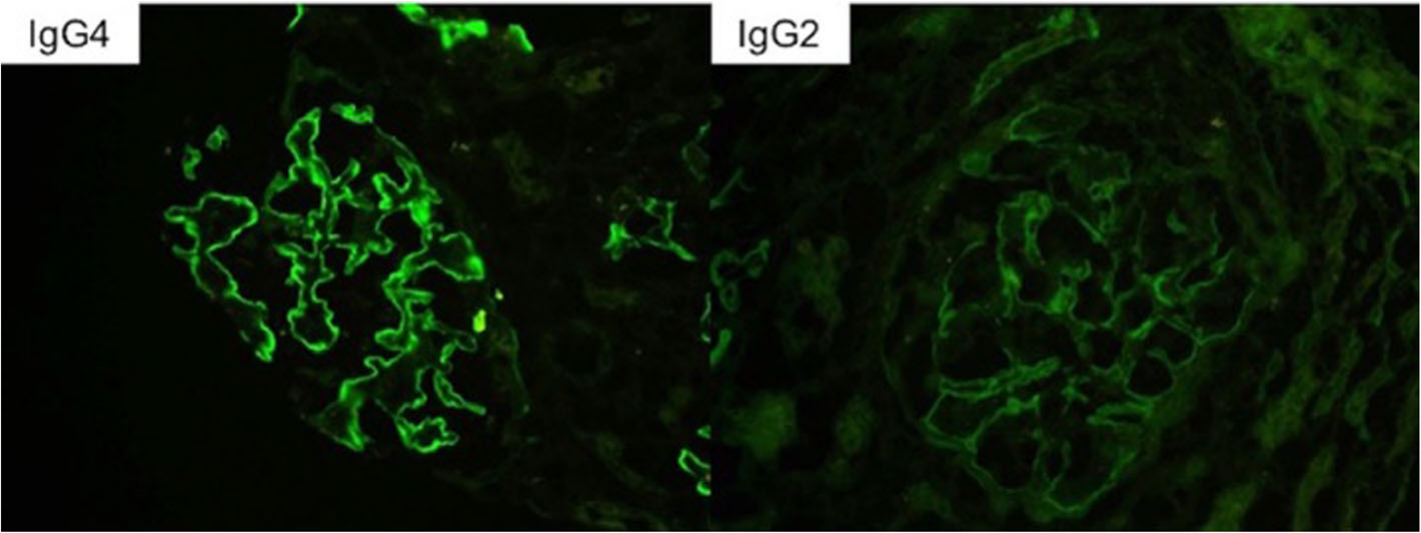
IF staining for IgG subtypes showed strong linear glomerular positivity for IgG4 and weaker staining for IgG2. IgG1 and IgG3 were negative, not shown. (magnification × 400)

Renal biopsy showed necrotizing and crescentic glomerulonephritis involving 70% of the glomeruli, diffuse endocapillary and mesangial hypercellularity and focal GBM duplication (Fig. 1). Direct IF microscopy showed strong linear IgG staining along the glomerular basement membranes and focal staining along tubular basement membranes. Linear staining was also observed for both light chains (lambda>kappa), with weaker IgA staining and no IgM, C3 or C1q staining (Fig. 2). Staining for IgG subtypes was positive for IgG2 and IgG4 in a linear pattern (IgG4 > IgG2) (Fig. 3). No immune complex-type dense deposits type nor “powdery” linear densities along the GBM were seen by electron microscopy (EM) (not shown).

Based on the combination of strong linear IgG staining in the glomeruli and circulating anti-GBM antibodies within normal range, a diagnosis of atypical anti-GBM disease was made, and the patient was started on high dose prednisone and IV cyclophosphamide. His hemoptysis was felt secondary to anti-GBM disease rather than hypervolemia from severe GN though he did not undergo bronchoscopy during that time. During workup, he was found to have elevated serum IgM lambda monoclonal protein at 1.5 g/L. On discharge, a chest x-ray showed resolution of the patchy infiltrates, and he was prescribed continuation of corticosteroids, IV cyclophosphamide 1.2 g biweekly [16] (received 4 treatments total), and trimethoprim/sulfamethoxazole for *Pneumocystis jiroveci* prophylaxis. Renal function also improved and at discharge his creatinine was 305 μmol/L, following a peak of 454 μmol/L.

**Fig. 4:**
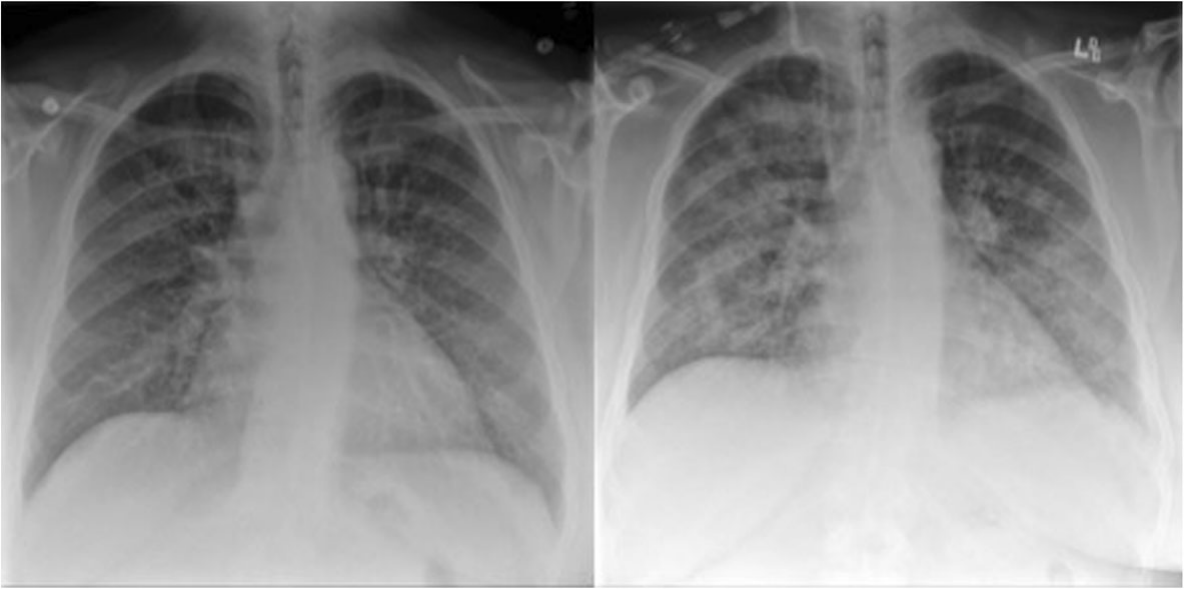
Left image shows the chest radiograph of the patient at initial presentation. Right image shows worsening of bilateral diffuse opacities at second presentation, found later to be related to CMV pneumonitis. Patient’s chest radiograph improved after initial presentation although not shown

The patient returned to Urgent Care approximately 1 month later with fever, massive hemoptysis, and anuria. Serum creatinine was 1065 μmol/L, and he was urgently started on dialysis. Chest X-ray showed worsening of bilateral patchy opacities (Fig. 4). Empirical treatment for presumed relapse of his disease was initiated, with 500 mg IV methylprednisolone and plasma exchange with fresh frozen plasma as replacement fluid. He also received empiric ceftriaxone and azithromycin and continued on prednisone 60 mg daily, along with cyclophosphamide, and trimethoprim/sulfamethoxazole. He underwent bronchoscopy, and his bronchoalveolar lavage did not initially reveal an infectious agent, although the respiratory panel was positive for parainfluenza. CMV was not assayed at the time of initial bronchoscopy. Of note, his BAL was persistently bloody but did not meet criteria for diffuse alveolar hemorrhage (his hemosiderin-laden macrophage count was < 20%).

The patient was managed for 5 days as above but continued to have massive hemoptysis and worsening pulmonary infiltrates radiographically. As a result, rituximab therapy was initiated for possible cyclophosphamide-resistant atypical-anti-GBM disease. The patient’s clinical condition continued to decline as he required intubation and underwent repeat bronchoscopy. He was empirically treated with piperacillin/tazobactam and linezolid despite all of his blood/alveolar lavage bacterial cultures being negative. His echocardiogram and enhanced chest CT were unremarkable for other etiologies contributing to severe pulmonary hemorrhage. He was subsequently started on empiric ganciclovir for possible cytomegalovirus (CMV) infection. Results of the second bronchoscopy confirmed CMV and a viral load of > 10^6^ IU/mL from his BAL fluid. Cytology showed inclusion bodies consistent with CMV infection. His serum showed a CMV load of > 2 million IU/mL. He never had CMV checked prior to this. After initiation of ganciclovir, the patient experienced clinical improvement with resolution of his hemoptysis and chest x-ray abnormalities. His renal function however never recovered, and he remained dialysis dependent.

## Discussion and conclusions

This is a case of a 24-year-old male with aggressive atypical anti-GBM disease who developed recurrent massive hemoptysis after initial apparent response to induction therapy with corticosteroids and cyclophosphamide, subsequently found to have CMV viremia and pneumonitis as the likely cause for his severe clinical deterioration. The atypical pathology with severe phenotype, undetectable anti-GBM serology, and presence of CMV pneumonitis are the three main highlights of this case.

To the best of our knowledge, this is the first case of atypical anti-GBM disease with a diffuse crescentic and endocapillary proliferative phenotype. Typical anti-GBM disease is associated with diffuse crescent formation without endocapillary or mesangial proliferation. [10, 13]. On the other hand, a series of 20 atypical anti-GBM cases reported by Nasr et al. [10] showed endocapillary and mesangial proliferation, or membranoproliferative glomerulonephritis. Crescents, when present, involved a minority of the glomeruli. In this series, patients tended to have a better renal outcome than typical anti-GBM and did not have pulmonary involvement, indicating a more benign illness [10]. Our case appears to deviate from this spectrum, with an aggressive renal disease course and pulmonary involvement, more akin to typical anti-GBM disease.

There are several possible reasons why histologically confirmed anti-GBM disease may be serologically negative by commercial immunoassays. One possible explanation is that the target antigen may be different, and thus the anti-GBM antibody is not detected by conventional laboratory immunoassays. An alternate antigen is also postulated to trigger alternate pathogenic pathways that result in a different pathologic appearance to the glomeruli [2, 10].

Standard anti-GBM enzyme-linked immunosorbent assays (ELISAs) are considered highly sensitive and specific. However, they are designed to detect IgG, and may give negative results when circulating anti-GBM antibodies are IgA class. In addition, standard anti-GBM assays may be less sensitive for certain IgG subclasses such as IgG4 [7, 12]. A recent report by Ohlsson et al. [17] noted atypical anti-GBM cases with relatively mild renal disease where the initial serology is borderline/negative for anti-GBM, but further testing specific for IgG4 anti-GBM ELISA showed positive results, supporting the concern for false negative reporting for cases with alternate IgG subclasses. IgG anti-GBM subclasses were not performed until retrospective analysis, and at that point the patient was immunosuppressed and they were low. However, IgG subtyping by IF showed strong positive staining for IgG4 and weaker staining for IgG2. It has been reported that IgG4 may cross react with IgG2 particularly in those individuals with high IgG4 [18].

Our chemiluminescence assay (BioFlash, Inova Diagnostics, San Diego, CA) used a fluorochrome-coupled monoclonal anti-human IgG as the secondary antibody which has been shown to bind all subclasses of IgG but has highest sensitivity for IgG1 and IgG3 (manufacturer’s communication). Our patient may therefore have had anti-GBM IgG4 at a level undetectable by the assay despite being pathogenic, although the limited capacity of IgG4 to bind complement and engage Fc receptors on neutrophils raises questions regarding their pathogenic potential [17].

This is a possible reason why serum anti-GBM remained negative throughout this patient’s course. The severe phenotype in association with anti-GBM IgG4 is unexpected in view of previous reports of mild disease associated with this subclass [17, 19] . Interestingly, Zhao et al. [20] found anti-GBM antibodies limited to IgG2 and IgG4 in the sera of normal patients and anti-GBM disease patients with normal renal function. However, they reported that a worse clinical outcome was linked to the presence and increased titers of IgG1 and IgG3 [20]. Similarly, the 4 cases with IgG4 anti-GBM serology reported by Ohlsson et al. [17] also showed favorable renal outcome, which was not the case in our patient.

The atypical renal pathology in our case, along with the IgM lambda monoclonal peak raised the possibility of proliferative glomerulonephritis with monoclonal immunoglobulin deposits (PGNMID) as well as Randall type monoclonal immunoglobulin deposition disease (MIDD). PGNMID and MIDD were ruled out as EM did not show any immune complex-type dense deposits nor ‘powdery’ linear densities along the GBM [21, 22],and by the fact that IF showed polytypic deposits, staining for both kappa and lambda and for two IgG subclasses, making it unlikely that the process was driven by the IgM lambda monoclonal gammopathy. The nodular mesangial sclerosis was quite striking, and diabetic nephropathy and idiopathic nodular glomerulosclerosis (ING) were considered. The patient had no previous diagnosis of diabetes and his most recent HbA1c was 5.6%, essentially excluding the likelihood of diabetic nephropathy. Batal et al. [23] describe 3 heavy smokers, none with diabetes, with nodular glomerulosclerosis with anti-GBM-like glomerulonephritis. Pulmonary hemorrhage was not a feature in any of the patients. On renal biopsy, they all showed strong linear GBM staining for IgG by IF, but were negative for anti-GBM by ELISA, although one was positive by immunoblotting. Notably, light microscopy did not show endocapillary proliferation but focal cellular and fibrocellular crescents were present, along with features most consistent with ING [23]. Ultimately, the endocapillary hypercellularity and cellular crescents seen in our case, without convincing mesangiolysis, microaneurysms, or Kimmelstiel-Wilson nodules as described in this small series, helped us reject ING as a diagnosis; however, it was a deliberation considering his extensive smoking history.

Donaghy and Rees [24] noted a close correlation between smoking and anti-GBM related pulmonary hemorrhage and relapse of pulmonary symptoms on re-exposure to cigarette smoke. Although it is unclear whether our patient continued to smoke marijuana in between his relapses, this may have contributed to his clinical presentation.

Secondly, to our knowledge, there are only two case reports that highlight the scenario of presumed relapse of anti-GBM disease, later discovered to be CMV induced pulmonary hemorrhage [14, 15]. Chan et al. [14] and Hasegawa et al. [15] noted that patients initially responded to immunosuppression, but subsequently developed relapse of pulmonary hemorrhage, initially considered to be relapse of their anti-GBM disease. Chan et al. [14] hypothesized that the CMV infection might have precipitated the relapse of the disease and the ultimately fatal pulmonary hemorrhage in his patient. Hasegawa et al. [15] reported a patient with anti-GBM disease who initially responded to immunosuppression, and on relapse was found to have CMV pneumonia which responded to anti-viral therapy. Although there are previous reports of anti-GBM relapse hypothesized to be induced by Influenza A [25] and bacterial infections [26], possibly due to infection causing enhancement of antibody mediated tissue injury, [14] it is more likely that our patient’s decompensation was secondary to opportunistic CMV pneumonitis rather than CMV induced anti-GBM disease relapse. The prompt response to ganciclovir and the failure to improve with plasmapheresis/steroids/rituximab supports this notion. It is unclear whether this would have been a primary CMV infection or reactivation of latent virus from immunosuppression. It is also unclear how the CMV infection contributed to the progression to the end-stage renal disease, however, the EULAR [16] treatment protocol is well established, hence emphasizing the importance of recognizing opportunistic CMV infection during apparent treatment failure.

This patient’s presentation with endocapillary hypercellularity, segmental membranoproliferative features, linear GBM staining for IgG by IF, and negative anti-GBM serology fits previous descriptions of “atypical” anti-GBM disease. [10] It is, however, unusual in that there were diffuse crescents and the patient developed pulmonary hemorrhage (both of which are generally seen in typical anti-GBM disease). The clinical behaviour was more aggressive than what is reported in atypical anti-GBM disease [10, 20, 22]. Typically, anti-GBM titers can be used as a guide to determine treatment length and when a transplant is deemed safe, an option not available when serology remains undetectable. Moreover, typical anti-GBM has a monophasic course, whereas variable rates of relapses have been reported in atypical anti-GBM cases, suggesting continuous management rather than tapering immunosuppression may need to be considered [8, 11]. Relapses are also more difficult to diagnose in the absence of positive serology, and in this case, the importance of recognizing CMV pneumonitis as a potential complication of immunosuppression cannot be overemphasized.

## Abbreviations

ALBIA: addressable laser bead immunoassay
Anti-GBM: anti-glomerular-basement membrane
BAL: bronchoalveolar lavage
CMV: cytomegalovirus
ELISAs: enzyme linked immunosorbent assays
EM: electron microscopy
GN: glomerulonephritis
IF: immunofluorescence
ING: idiopathic nodular glomerulosclerosis
MIDD: monoclonal immunoglobulin deposition disease
PGNMID: proliferative glomerulonephritis with monoclonal immunoglobulin deposits
